# Quantitative Evaluation System of Soft Neurological Signs for Children with Attention Deficit Hyperactivity Disorder

**DOI:** 10.3390/s16010116

**Published:** 2016-01-18

**Authors:** Miki Kaneko, Yushiro Yamashita, Keiji Iramina

**Affiliations:** 1 Graduate School of Systems Life Sciences, Kyushu University, 3-1-1 Maidashi, Higashi-Ku, Fukuoka-Shi, Fukuoka 812-8582, Japan; iramina@inf.kyushu-u.ac.jp; 2 Department of Pediatrics and Child Health, Kurume University School of Medicine, 67 Asahi-Machi, Kurume-Shi, Fukuoka 830-0011, Japan; yushiro@med.kurume-u.ac.jp; 3 Faculty of Information Science and Electrical Engineering, Kyushu University, 744 Motooka, Nishi-Ku, Fukuoka-Shi, Fukuoka 819-0395, Japan

**Keywords:** acceleration and angular velocity sensors, motion analysis, soft neurological signs, pronation, supination, attention deficit hyperactivity disorder

## Abstract

Attention deficit hyperactivity disorder (ADHD) is a neurodevelopmental disorder characterized by symptoms of inattention, hyperactivity, and impulsivity. Soft neurological signs (SNS) are minor neurological abnormalities in motor performance, and are used as one evaluation method for neurodevelopmental delays in children with ADHD. Our aim is to establish a quantitative evaluation system for children with ADHD. We focused on the arm movement called pronation and supination, which is one such soft neurological sign. Thirty three children with ADHD aged 7–11 years (27 males, six females) and twenty five adults participants aged 21–29 years old (19 males, six females) participated in our experiments. Our results suggested that the pronation and supination function in children with ADHD has a tendency to lag behind that of typically developing children by several years. From these results, our system has a possibility to objectively evaluate the neurodevelopmental delay of children with ADHD.

## 1. Introduction

The current diagnostic methods for neurodevelopmental disorders are based on pre/perinatal, developmental, family history, and physical/neurological examination, including visual inspection of so-called soft neurological signs (SNS). SNS are minor neurological motor performance abnormalities used as findings to test for developmental delays in neurological function [1]. SNS have been associated with developmental disorders presenting as difficulties in behavior, coordination, and learning. If neurodevelopmental function is impaired or delayed, soft neurological signs appear in various forms during physical examinations. Evaluation of physical functioning in children is therefore very useful for diagnosing developmental disorders from an early stage. Currently, most SNS development tests are used as a form of visual observation by pediatricians based on the several criteria for visual observation [1,2]. However this approach is not a quantitative method. 

Previous studies have reported the differences between typically developing (TD) children and several developmental disorders: attention deficit hyperactivity disorder (ADHD) [3,4,5,6,7,8,9], learning disorder (LD) [10], autism spectrum disorder (ASD) [11,12], developmental coordination disorder [9,13,14] in response time and evaluation score by these criteria. However, there is little information to quantify the SNS method itself for developmental disorders. It is necessary to establish criteria for a more quantitative evaluation. Quantifying SNS are important for detecting developmental disorders and providing appropriate educational support from an early stage. 

We focused on pronation and supination which is one of the SNS test methods. Previous studies have quantitatively measured this test [15,16,17], however, the target of those studies was not developmental disorders. There is little information to establish quantitative evaluation system for developmental disorders. 

In our previous paper, we reported a quantitative evaluation system using acceleration and angular velocity sensors [18]. We measured typically developing (TD) children using this system. From measurement data of TD children, we could obtain the developmental curves of pronation and supination [19]. The results show that our system could become quantitative criteria for children with developmental disorders. 

In this study, our aim was to establish a quantitative evaluation system of soft neurological signs for children with ADHD. For this purpose we focused on ADHD which is one such developmental disorder. We measured 33 children aged 7–11 years with ADHD (27 males, six females) and 25 adults aged 21–29 years (19 meals, six females) and looked into the characteristics of ADHD. 

## 2. Systems Design Section

### 2.1. System Configuration and Procedure of Experiment

**Figure 1:**
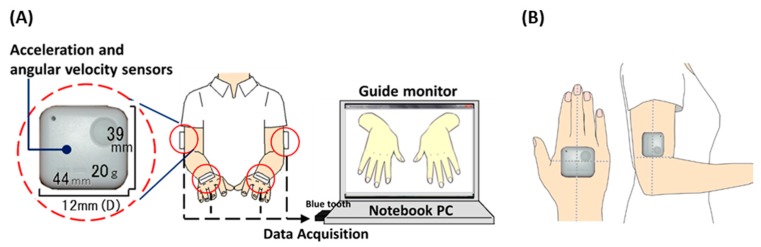
System configuration (**A**) and the position of sensors (**B**).

Our system is comprised of four acceleration and angular velocity sensors (WAA-006, WAA-010, ATR-Promotions, Kyoto, Japan), a guide monitor (CLAiR SK-DTV 133JW2, Sknet, Kanagawa, Japan) and a notebook PC (VAIO VGN-NW91FS, Sony, Tokyo, Japan). The subject were attached these sensors on both hands and elbows, as shown in Figure 1.

In accordance with observation items for developmental disorders, in this research we focused on two motor tasks: an imitative motor task and a maximal-effort motor task performed with only one hand. Measurement time was 10 s for each task. When the participants performed the imitative motor task, they imitated the motion on a guide monitor. The hands on guide monitor test was performed at a rate of 80 times per minute (about 1.3 Hz). In the maximal-effort motor task, the participants moved their hand as fast as possible. The participants were instructed to maintain their non-rotating hand in a horizontal position, as shown in Figure 1.

### 2.2. Participants

Thirty three children with ADHD aged 7–11 years (27 males, six females) and twenty five adults participants aged 21–29 years old (19 males, six females) participated in our experiment (Table 1). Children with ADHD were recruited from Kurume University Hospital. They were diagnosed with ADHD by The Diagnostic and Statistical Manual of Mental Disorders, Fourth Edition (DSM-IV) and had an Intelligence Quotient (IQ) of more than 70 as defined by WISC-III. 

**Table 1:** Descriptive data of study participants.

TD Group				ADHD Group			
Age	Male/Female	Total		Age	Male/Female	Total	IQ
4	6/3	9					
5	9/5	14					
6	4/7	11					
7	13/19	32		7	4/2	6	110.8 (10.9)
8	22/14	36		8	3/1	4	101.8 (20.7)
9	19/21	40		9	8/2	10	94.0 (9.0)
10	17/20	37		10	5/1	6	99.4 (6.2)
11	8/18	26		11	7/0	7	103.9 (10.4)
12	9/9	18					
21–29	19/6	25					
Total	126/122	248		Total	27/6	33	101.3 (12.9)

SD in parentheses. IQ was assessed using the Wechsler Intelligence Scale for Children-Third Edition (WISC-III).

All children and their parents received an explanation of the aim, and the procedures and hazards of the experiment before our measurement. They all agreed to participate. The participants attended the first experimental session. The study was approved by the Kyushu University Ethics Committee.

## 3. Analysis Design

### 3.1. Evaluation Indices and Analyzed Parameters

There are four observation indices to evaluate pronation and supination: (a) speed; (b) associated movement; (c) elbow excursion, and (d) pauses at extreme positions of the pronated and supinated hand [1,2]. Associated movement during pronation and supination is movement which manifests itself in the contralateral hand by associated pronation and supination [1]. In accordance with these observation items for developmental disorder, we quantified these four indices using data obtained from acceleration and angular sensors (hereinafter called rotational speed, mirror movement, postural stability of rotating elbow and temporal change of rotational size in each index). 

Rotational speed used the peak frequency of acceleration on the Z axis in the continuous fast Fourier transform (FFT). Mirror movements used the absolute value total sum of acceleration and angular velocity on the Z axis and X axis. Postural stability of rotating elbows was calculated using the absolute value total sum of acceleration on the Z axis. Measurement time per a task is 10 s. In this study, we separated seven phases in the measurement waveform (2.5 s per a phase; overlap, 1.25 s) to analyze the temporal changes of measurement time. Therefore the value of a parameter was mean value of seven phases. Temporal change of rotational size is an index to evaluate whether the child can accurately pronate and supinate at a fixed rotational angle. Temporal change of rotational size used the variance in peak frequency’s power of the continuous FFT among the seven phases (10 s).

Moreover, to compare between our previous results of typically developing children and data of children with ADHD, we used other two indices: bimanual symmetry and compliance. Bimanual symmetry is an evaluation index of whether the participant’s left hand and right hand move in symmetry or not. This index used the correlation coefficient of acceleration on the Z axis, and phase difference of acceleration waveform on the Z axis between the both hands. Compliance is an evaluation index of whether the motion speed between the subject’s hands and the hands on the guide monitor is the same or not. This index used the difference between about 1.3 Hz (rotational speed of the hands on the guide monitor) and the rotational speed of the subject’s hands. The sampling frequency was 100 Hz. A 6 Hz low-pass was applied to the signal.

### 3.2. Score and Statistics for Evaluation Indices

Analyzed parameters discussed in the above were normalized by the data of participants aged 21–29 years old using the following formula:

$$y_{h}=80+\frac{10}{\sigma_{a}}(-x_{h}+\mu_{a})$$

The *x_h_* is the analyzed value in all participants. The *μ_a_* and *σ_a_* are the mean value and standard deviation of the each analyzed values in participants aged 21–29 years old. The *y_h_* indicate score of pronation and supination in each indices. To compare between TD children and children with ADHD in same age group, we used the Mann-Whitney U test for statistics. 

## 4. Results

**Figure 2:**
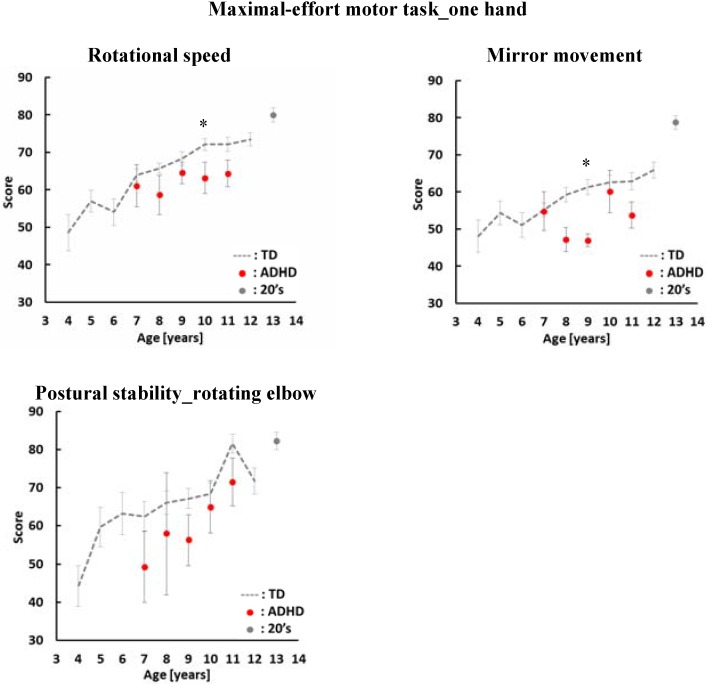
Average scores and standard errors in the maximal-effort motor task. The average score of children with ADHD is indicated by the red dots. The gray dashed line shows the average score of TD children. Gray dots show the average scores of adults aged 21–29 years old. *: *p* < 0.05.

**Figure 3:**
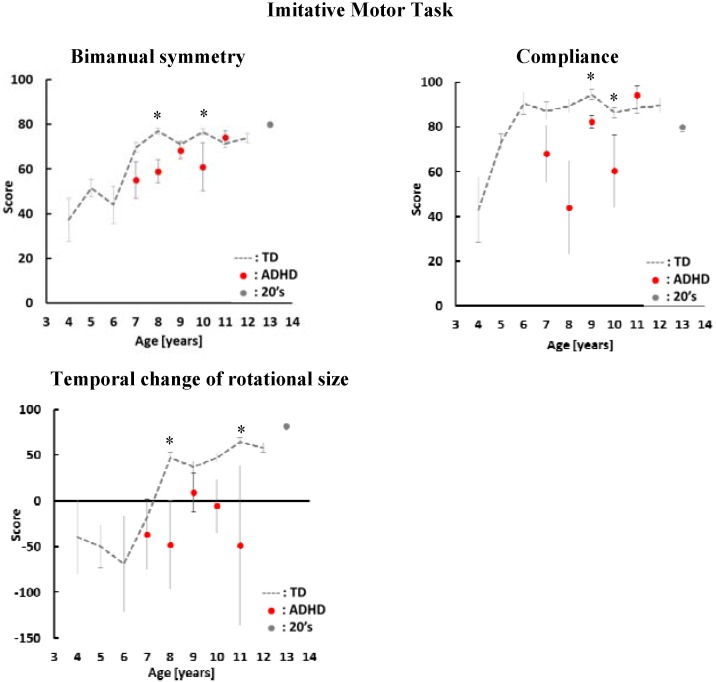
Average scores and standard errors in the imitative motor task. The average score of children with ADHD is indicated by the red dots. The gray dashed line shows the average score of TD children. Gray dots show the average scores of adults aged 21–29 years old. *: *p* < 0.05

Figure 2 and Figure 3 show the average score of ADHD children by age. The vertical axis and the horizontal axis show the evaluation index score and the age of the participants. The average score of children with ADHD is indicated by the the red dots. The gray dashed line shows the average score of TD children. Gray dots shows the average score of adults. In this study we reanalyzed TD children data using the same conditions of the ADHD children’s data to compare the differences between TD children and children with ADHD.

In our previous study, we could obtain the developmental curves of pronation and supination in TD children using our evaluation system. Therefore we looked into the characteristics of children with ADHD and the difference between TD children and children with ADHD by age in this study.

In the one hand maximal-effort motor task, we have three evaluation indices: rotational speed, mirror movement and postural stability of elbow. As shown Figure 2, the scores of children with ADHD in all indices have a tendency to be lower than the scores of TD children. There was a significant difference between TD children and children with ADHD aged 10 years old in rotational speed. In mirror movement, there was a significant difference between TD children and children with ADHD aged 9 years old. On the other hand, there was no significant difference between TD children and children ADHD in postural stability of the rotation elbow.

In case of TD children, the score increased as they grew older. In rotational speed and postural stability of rotating elbow, the score of ADHD increased the same as TD children with age. On the other hand, there was no relationship between age and score of mirror movement in children with ADHD. 

In the imitative motor task, we have three indices: bimanual symmetry, compliance and temporal change. As shown Figure 3, the scores of children with ADHD in all indices have a tendency to be lower than the scores of TD children. There were significant differences between TD children and children with ADHD aged 8 and 10 years old in bimanual symmetry. In the temporal change of rotational size, there was a significant difference between TD children and children with ADHD aged 8 and 11 years old. In compliance, there was significant difference between TD children and children with ADHD aged 9 and 10 years old. 

In bimanual symmetry, the score of children with ADHD increased with age. On the other hand, the variability of ADHD scores was larger than the variability of TD children’s score in compliance and temporal change of rotational size. There was no relationship between age and the score of children with ADHD in these indices. 

**Figure 4:**
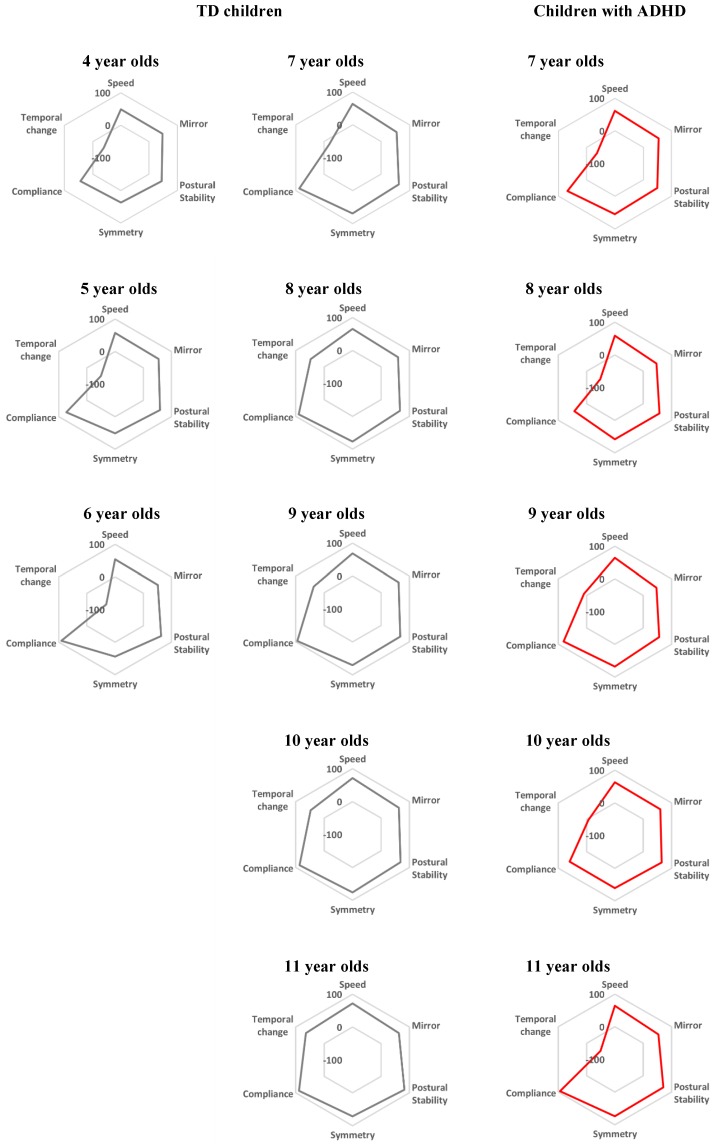
The comparison of function’s balance between TD children and children with ADHD in pronation and supination. Speed, Rotational speed; Mirror, Mirror movement; Postural stability, Postural stability of elbow; Symmetry, Bimanual symmetry; Temporal change, Temporal change of rotational size. Gray radar charts show the data of TD children. Red radar charts show the results for children with ADHD.

Figure 4 shows the comparison of function’s balance in pronation and supination between TD children and children with ADHD. Speed, Mirror, Postural stability, Symmetry and Temporal change in Figure 4 show each evaluation index: rotational speed, mirror movement, postural stability of elbow, bimanual balance of TD children in each age. Red radar charts show the function’s balance of children with ADHD in each age. As shown in Figure 4, the function’s balance of children with ADHD is similar to the function’s balance of TD children who are younger than the age of the children with ADHD.

## 5. Discussion

Pronation and supination is SNS test for children of 4 years and older. The function of pronation and supination improves as children grow. Mirror movement during pronation and supination is movement which manifests itself in the contralateral hand by associated pronation and supination [1]. This movement decreases as TD children grow older [20]. Our results fromTD children also indicated that the pronation and supination function in TD children improved as they grew older. Our results are consistent with the report.

On the other hand, the function of pronation and supination was lower than the function of TD children. In rotational speed, postural stability of rotating elbow and bimanual symmetry, the pronation and supination function in children with ADHD has a tendency to develop several years late. Moreover, the function’s balance of children with ADHD is similar to the function’s balance of TD children who are younger than the age of the children with ADHD. Previous studies reported that cortical development of children with ADHD lagged behind that of TD children by several years [21]. Our results are consistent with that report. Therefore, our results show that our system could become a quantitative criterion to evaluate developmental delays in neurological function for children with ADHD.

However, there was no relationship between age and score of children with ADHD in mirror movement, compliance and temporal change of rotational size. Previous research on functional magnetic resonance imaging (MRI) reported activation of the ipsilateral cerebellum in typically developing adults during performing pronation and supination [22]. Moreover, the cerebellum was associated with ADHD [23].

In this study, we indicated that the pronation and supination in TD children and children with ADHD was only associated with age-appropriate development. We could not demonstrate whether our proposed method using acceleration and angular velocity sensors alone was useful for distinguishing children with ADHD from TD. We surmise that the pronation and supination may be associated not only with age but also differences of severity of ADHD or subtypes of ADHD: combined-type, inattentive-type, and hyperactive-impulsive-type. In this study, the number of children with ADHD was not enough to compare the differences in pronation and supination by severity of ADHD symptoms or by subtypes of ADHD. We need to measure more participants to answer this question in the future. 

SNS evaluation provides information about neurological conditions concerning motor function, learning, and behavioral problems [1]. However, children with ADHD usually have multiple problems such as motor coordination, learning, and cognition. SNS is one method for evaluating children with developmental delay. We need to consider the correlation between the function of pronation/supination and the Developmental Coordination Disorder Questionnaire (DCDQ) used for the screening developmental coordination disorders [24]. We also need to look into relationships between severity, subtypes of ADHD and the function of pronation and supination, and then we might be able to systemically evaluate children using methods sensitive to each severity or subtype of ADHD in the future. 

## 6. Conclusions

In this study, we quantified some characteristics of ADHD children. From a comparison between TD children and children with ADHD by age, the pronation and supination function in children with ADHD has a tendency to lag behind that of typically developing children by several years. In conclusion, our system can be applied to evaluate the neurodevelopmental delay of children with ADHD. In the future, we plan to look into relationships between the pronation and supination function and the symptoms of different ADHD sub-types: combined-type, inattentive-type, and hyperactive-impulsive-type.

## References

[1] Mijna H.A. (2010). The Neurological Examination of the Child with Minor Neurological Dysfunction.

[2] Touwen B.C.L., Prechtl H.F.R. (1970). The Neurological Examination of the Child with Minor Neurological Dysfunction.

[3] Patankar C.V., Sangle J.P., Shah R.H., Dave M., Kamath M.R. (2012). Neurological soft signs in children with attention deficit hyperactivity disorder. Indian J. Psychiatry.

[4] Fewell R.R. (2002). Attention Deficit Hyperactivity Disorder in Very Young Children: Early Signs and Interventons. Inf. Young Child..

[5] Uslu R., Kapci G.E., Oztop D. (2007). Neurological soft signs in comorbid learning and attention deficit hyperactivity disorders. Turk. J. Pediatr..

[6] Pineda D., Ardila A., Rosselli M., Arias E.B., Henao C.G., Gomez F.L., Mejia E.S., Miranda L.M. (1999). Prevalence of Attention-Deficit/Hyperactivity Disorder Symptoms in 4- to 17-Year-Old Children in the General Population. J. Abnorm. Child Psychol..

[7] Gonga J., Xie J., Chenb G., Zhanga Y., Wang S. (2015). Neurological soft signs in children with attention deficit hyperactivity disorder: Their relationship to executive function and parental neurological soft signs. Psychiatry Res..

[8] Kroes M., Kessels A.G.H., Kalff A.C., Feron F.J.M., Vissers Y.L.J., Jolles J., Vles J.S.H. (2002). Quality of movement as predictor of ADHD: Results from a prospective population study in 5- and 6-year-old children. Dev. Med. Child Neurol..

[9] Williams J., Omizzolo C., Galea M.P., Vance A. (2013). Motor imagery skills of children with Attention Deficit Hyperactivity Disorder and Developmental Coordination Disorder. Hum.s Mov. Sci..

[10] Adams R.M., Kocsis J.J., Estes R.E. (1974). Soft neurological signs in learning- disabled children and controls. Am. J. Dis. Child..

[11] De Jong M., Punt M., de Groot E., Minderaa R.B., Hadders-Algra M. (2011). Minor neurological dysfunction in children with autism spectrum disorder. Dev. Med. Child Neurol..

[12] Williams J., Thomas P.R., Maruff P., Butson M., Wilson P.H. (2006). Motor, visual and egocentric transformations in children with Developmental Coordination Disorder. Child Care Health Dev..

[13] Wilmut K., Wann J.P., Brown J.H. (2006). Problems in the coupling of eye and hand in the sequential movements of children with Developmental Coordination Disorder. Child Care Health Dev..

[14] Freitag C.M., Kleser C., Schneider M., Gontard A.V. (2007). Quantitative Assessment of Neuromotor Function in Adolescents with High Functioning Autism and Asperger Syndrome. J. Autism Dev. Disord..

[15] Kreulen M., Smeulders M.J.C., Veeger H.E.J., Hage J.J., Vanderhorst C.M.M. (2004). Three-dimensional video analysis of forearm rotation before and after combined pronator teres rerouting and flexor carpi ulnaris tendon transfer surgery in patients with cerebral palsy. J. Hand Surg. Br. Eur..

[16] Okada M., Okada M. (1983). A method for quantification of alternate pronation and supination for forearms. Comput. Biomed. Res..

[17] Hermsdorfer J. (1999). Comparative analysis of diadochokinetic movements. J. Electromyogr. Kinesiol..

[18] Iramina K., Kamei Y., Katayama Y. Evaluation System for Minor Nervous Dysfunction by Pronation and Supination of Forearm using Wireless Acceleration and Angular Velocity Sensors. Proceedings of the 2011 Annual International Conference of the IEEE Engineering in Medicine and Biology Society (EMBC).

[19] Kaneko M., Yamashita Y., Inomoto O., Iramina K. (2015). Soft Neurological Signs in Childhood by Measurement of Arm Movements Using Acceleration and Angular Velocity Sensors. Sensors.

[20] Largo R.H., Caflish J.A., Hug F., Muggli K., Monar A.A. (2001). Neuromotor development from 5 to 18 years. Part 2: Associated movements. Dev. Med. Child Neurol..

[21] Landgren M., Kjellman B., Gillberg C. (2000). Deficits in attention, motor control and perception (DAMP): A simplified school entry examination. Acta Paediatr..

[22] Tracy J.I., Faro S.S., Mohammed F.B., Pinus A.B., Madi S.M., Laskas J.W. (2001). Cerebellar mediation of the complexity of bimanual compared to unimanual movement. Neurology.

[23] Hoppenbrouwers S.S., Schutter D.J., Fitzgerald P.B., Chen R., Daskalakis S.K. (2008). The role of the cerebellum in the pathophysiology and treatment of neuropsychiatric disorders: A review. Brain Res. Rev..

[24] Wilson B.N., Kaplan J.B., Crawford G.S., Campbell A., Dewey D. (2000). Reliability and Validity of a Parent Questionnaire on Childhood Motor Skills. Am. J. Occup. Ther..

